
JOURNAL INFORMATION
==============================
NLM Title Abbreviation: Wirtschaftsdienst
Journal ID: Wirtschaftsdienst
NLM Journal ID: 101086612

Wirtschaftsdienst (Hamburg, Germany : 1949)

ISSN: 0043-6275

ARTICLE INFORMATION
==============================
PMCID: PMC8383022
PMID: 34456384
DOI: 10.1007/s10273-021-2976-4
Article ID: 2976
Article version: 1
Subjects: Zeitgespräch

Besseres Marktdesign im Gesundheitswesen

Wambach Achim achim.wambach@zew.de

grid.13414.33 0000 0004 0492 4665 Wirtschaftsforschung GmbH Mannheim, ZEW - Leibniz-Zentrum für Europäische, L 7, 1, 68161 Mannheim, Deutschland
Electronic publication date: 2021 Aug 24
Print publication date: 2021
Volume: 101
Issue: 8
First page: 590
Last page: 593
Copyright: © Der/die Autor:in(nen) 2021
License: Open Access: Dieser Artikel wird unter der Creative Commons Namensnennung 4.0 International Lizenz veröffentlicht (https://creativecommons.org/licenses/by/4.0/deed.de).
Open Access wird durch die ZBW — Leibniz-Informationszentrum Wirtschaft gefördert.
License URL: https://creativecommons.org/licenses/by/4.0/

issue-copyright-statement: © The Author(s) 2021

==============================

Die Corona-Krise hat in vielen Teilen der Wirtschaft Defizite offengelegt, aber auch neue Möglichkeiten eröffnet, so auch im Gesundheitswesen. Lernen aus der Krise beinhaltet, die Defizite kritisch zu analysieren und die neuen Chancen zu nutzen. Dafür sind auch institutionelle Reformen notwendig.

## Defizite in der Digitalisierung des Gesundheitswesens

Deutschland wäre besser durch die Krise gekommen, wenn das Gesundheitssystem weiter digitalisiert gewesen wäre, als es derzeit ist. So konstatiert der Wissenschaftliche Beirat beim Bundeswirtschaftsministerium in seinem aktuellen Gutachten (Wissenschaftlicher Beirat beim BMWI, 2021), dass die Digitalisierung des deutschen Gesundheitssystems derjenigen in anderen europäischen Ländern hinterherhinke. Die Erkenntnis ist nicht neu: Eine Studie von 2017 (Graumann und Bertschek, 2017), vergleicht die verschiedenen Branchen in Bezug auf die Digitalisierung und kommt zu dem Ergebnis, dass gerade das Gesundheitswesen nur niedrig digitalisiert ist (vgl. Abbildung 1). Die Kosten der mangelhaften Digitalisierung in der Krise sind hoch. So zeigen Studien aus der Finanzkrise (z. B. Bertschek et al., 2017) dass Unternehmen, die stärker digitalisiert sind, krisenfester sind. Erste Analysen deuten darauf hin, dass in der Corona-Krise Ähnliches gilt.

## Chancen durch neue Anwendungen des Marktdesigns

Die Krise und der dadurch erzeugte Handlungsdruck haben neue Möglichkeiten offenbart und zu einer Diskussion um neue Instrumente geführt. Einige Beiträge in diesem Zeitgespräch zeugen davon. Gerade die Möglichkeiten des Marktdesigns können noch vertiefter im Gesundheitssystem angewandt werden, nicht nur in der Pandemie.

Abbildung 1
**Digitalisierung nach Branchen**
Index = MAX 100

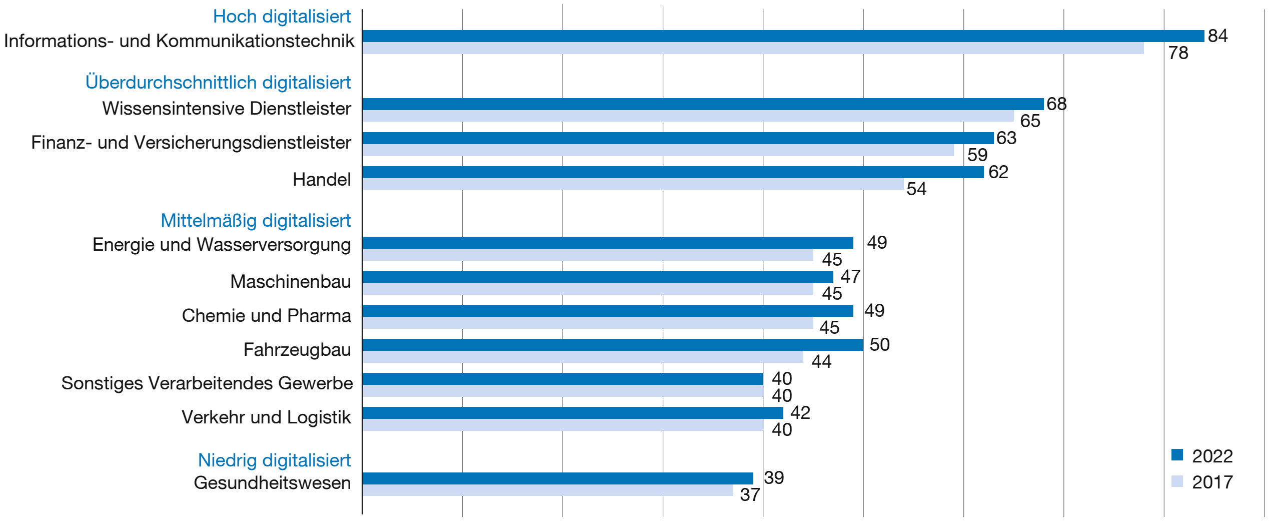

Quelle: Monitoring-Report Wirtschaft DIGITAL 2017. Repräsentative Unternehmensbefragung, für 2022 Erwartungswerte, eigene Darstellung.

## Marktdesign zur Innovationsförderung und zum Aufbau von Produktionsstätten

Die unglaublich schnelle Entwicklung von Impfstoffen ist eine Erfolgsgeschichte dieser Krise. Der Rollout der Impfstoffproduktionsstätten hätte zwar noch besser erfolgen können, aber auch hier wurden die anfänglichen Erwartungen übertroffen. Mit dazu beigetragen haben die sogenannten Push- und Pull-Verträge mit der Pharmaindustrie, die insbesondere in den USA verwendet wurden und die auf Anregungen von Marktdesigner:innen zurückgehen (Athey et al., 2020; Castillo et al., 2021; Fabra et al., 2020; Gretschko und Wambach, 2021). Dabei wurden Teile der Forschungsaufwendungen übernommen, Impfstoffproduktionsstätten bereits vor der Zulassung des Impfstoffs mitfinanziert, und eine Zahlung für den erfolgreichen Impfstoff in Aussicht gestellt. Methodisch sind diese Verträge eine Weiterentwicklung der sogenannten Advance Market Commitments, die erstmals 2007 für die Entwicklung neuer Pneumokokken-Impfstoffe zur Anwendung in Entwicklungsländern eingesetzt wurden (Kremer et al., 2020). Wie der Name andeutet, wird hierbei durch die garantierte Abnahme ein Markt für investitionsintensive Produkte geschaffen.

Die Verwendung dieses Instruments könnte man sich auch im deutschen Gesundheitssystem vorstellen, an Stellen, wo Unternehmen die Leistung nicht eigenständig wirtschaftlich erbringen können. Dies ist z. B. bei der Sicherstellung der regionalen Versorgung durch Ärzt:innen und Apotheken der Fall (Monopolkommission, 2018).

## Marktdesign zur Bereitstellung von Kapazitäten für den Einsatz in Krisenzeiten

Eine klassische Anwendung von Marktdesign ist die Sicherung der Bereitstellung von Kapazitäten. Dieses Instrument der „Kapazitätsmärkte“ wird vielfach im Strommarkt eingesetzt, wo verlässliche Stromerzeugungskapazitäten bereitgestellt werden müssen. So kann auch in Zeiten, in denen die Stromnachfrage sehr hoch ist oder in denen wenig Wind weht und der Himmel bewölkt ist, wo also ein Teil der erneuerbaren Energien keinen Strom produzieren kann, die Stromlieferung aufrecht erhalten werden (Cramton und Stoft, 2005). Ganz aktuell werden sogenannte Pandemie-Bereitschaftsverträge zur Sicherung der Impfstoffproduktionskapazität diskutiert. Der Beitrag von Axel Ockenfels in diesem Zeitgespräch beschäftigt sich damit. Eine wesentliche Herausforderung dabei ist, sicherzustellen, dass die Kapazität in der Krise dann auch einsatzfähig ist.

Auch dieses Instrument des Marktdesigns könnte weitere Anwendungen im deutschen Gesundheitssystem finden. Und zwar immer dort, wo Kapazitäten bereitgehalten werden sollen, die nur in besonderen Situationen zum Einsatz kommen. Neben der bereits erwähnten Produktionskapazität für Impfstoffe kämen auch die Vorhaltung von Krankenhausbetten, Intensivbetten oder medizinischen Geräten hierfür infrage.

## Marktdesign zur Zuteilung von knappen Ressourcen

Ein weiteres Thema in der Krise, mit dem sich Marktdesign beschäftigt hat, ist die Zuteilung von Impfstoffen sowohl an Menschen (der Beitrag von Dorothea Kübler in diesem Zeitgespräch beschäftigt sich damit; Kübler, 2021) wie auch an Intermediäre wie Arztpraxen (Gretschko und Ott, 2021). Solche Zuteilungsmärkte haben eine lange Tradition im Marktdesign. Alvin Roth, der später den Nobelpreis erhielt, beschäftigte sich schon in den 1980er Jahren mit der Zuteilung von Medizinstudierenden nach Ende ihres Studiums auf Kliniken (Roth, 1984). Ein aktuelleres Projekt beschäftigt sich damit, Lebensmittelspenden schnell und bedarfsorientiert an die Tafeln zu verteilen.[1] Den Projekten gemein ist, dass versucht wird, den Präferenzen beider Seiten gerecht zu werden, wie auch ein weiteres aktuelles Beispiel aus Großbritannien illustriert: Flüchtlinge werden dort nicht mehr ausschließlich nach Kriterien der Kommunen verteilt. Stattdessen wird nun auch auf ihre Präferenzen geachtet, sodass sie beispielsweise wahrscheinlicher in jene Gegenden kommen können, wo bereits Verwandte von ihnen wohnen (Delacrétaz et al., 2020; Gretschko, 2019).

Solche Zuteilungsmärkte spielen auch im deutschen Gesundheitssystem eine wichtige Rolle, und die Erkenntnisse des Marktdesigns könnten dort verstärkt genutzt werden. Und zwar immer dann, wenn es um eine Priorisierung knapper Ressourcen geht, wie z. B. bei der Verteilung von Intensivkapazitäten zwischen Krankenhäusern oder der Zuteilung von Spendernieren (Roth, 2004; Kübler und Ockenfels, 2020).

## Implikationen für das Gesundheitswesen

Aus den Fehlern lernen und die neuen Chancen ergreifen – das sollte die Konsequenz aus der Corona-Krise sein. Dies bedarf eines geeigneten institutionellen Rahmens. Die Studie des Wissenschaftlichen Beirats beim Bundeswirtschaftsministerium (2021) diagnostiziert ein Defizit in der Digitalisierung der öffentlichen Verwaltung, der allgemeinbildenden Schulen und des Gesundheitssystems. Das verwundert insofern, als dass im Gegensatz zu den anderen beiden Bereichen das Gesundheitssystem wettbewerblich aufgestellt ist. So stehen Krankenhäuser, Ärzteschaft und Krankenkassen jeweils im Wettbewerb untereinander. Dieser Wettbewerb agiert allerdings mit Handbremse: So wurde im März 2020 neu im Sozialgesetzbuch (SGB) V aufgenommen, dass „der Wettbewerb der Krankenkassen dazu dient, das Leistungsangebot und die Qualität zu verbessern und die Wirtschaftlichkeit zu erhöhen“ (§ 4a SGB V). Doch im Abschnitt davor ist ausgeführt, dass „im Interesse der Leistungsfähigkeit und Wirtschaftlichkeit der gesetzlichen Krankenversicherung die Krankenkassen miteinander arbeiten“ (§ 4 SGB V). Dass Krankenkassen gleichzeitig im Wettbewerb stehen und miteinander arbeiten sollen, ist nicht konsistent. Der „solidarische Wettbewerb“, der als Konzept der Wettbewerbsordnung für die GKV dient, stößt an Grenzen.

Eine Konsequenz dieses solidarischen Wettbewerbs ist der Risikostrukturausgleich (RSA), der in seiner derzeitigen Ausgestaltung allerdings dazu beiträgt, dass das Gesundheitssystem wenig innovationsfreudig ist (Monopolkommission, 2017). Der RSA regelt, welche Zahlungen eine gesetzliche Krankenkasse für ihre Versicherten erhält. Vereinfacht funktioniert dies so, dass gesetzlich Versicherte und Arbeitgeber:innen ihre Beiträge in einen Gesundheitsfonds einzahlen, der das Geld nach einem Verteilungsschlüssel des RSA an die Krankenkassen verteilt. Dieser Verteilungsschlüssel basiert auf dem Krankheits- bzw. Gesundheitszustand der jeweiligen Versicherten, und die Krankenkasse erhält die erwarteten Ausgaben des nächsten Jahres für jede versicherte Person erstattet.

Dieser „morbiditätsorientierte RSA“ ist für ein Versicherungssystem insofern konsistent, als dass die Krankenversicherer dadurch, dass sie die erwarteten und nicht die realisierten Ausgaben erstattet bekommen, Anreize bekommen, diese Ausgaben und natürlich ihre eigenen Verwaltungskosten zu senken. In der Umsetzung entstehen aber immer wieder Probleme, wie die Evaluierungsgutachten des Wissenschaftlichen Beirats zur Weiterentwicklung des Risikostrukturausgleichs von 2017 und 2018 dokumentieren (Drösler et al., 2017, 2018). Eine stetige Evaluierung und Anpassung des RSA ist daher notwendig und angemessen.

Diese reine Versicherungslogik des RSA greift aber dann nicht mehr, wenn die Krankenversicherer selber als Akteure im Gesundheitssystem tätig sind und die Art der Behandlung mitgestalten, anstatt ausschließlich die Kosten zu übernehmen. Die Kurzfristperspektive des RSA – es werden lediglich die erwarteten Ausgaben des Folgejahres erstattet – setzt sich dann fort in einer Kurzfristperspektive der Krankenkasse. Und diese Perspektive ist für den Einsatz von Innovationen, wie die Digitalisierung, aber auch für Prävention und langwirkende Behandlungen hinderlich, weil diese Maßnahmen Investitionen (also Ausgaben) zum heutigen Zeitpunkt bedeuten, deren Erfolge sich nicht schon im nächsten Jahr, sondern erst später in der Zukunft zeigen. Diese Erfolge werden dann nicht oder nicht ausreichend durch den RSA berücksichtigt.

## Was ist zu tun?

Die Gestaltung des Gesundheitswesens ist eine „Ewigkeitsaufgabe“, und vieles wurde bereits erreicht. Die Erfahrungen aus der Krise sollten Anlass geben, diesen Prozess weiter zu verfolgen. Die Analyse ist dabei der

1 Mehr zum Projekt unter: https://www.tafel.de/projekte/tafel-macht-zukunft-gemeinsam-digital/.

erste Schritt. Das deutsche Gesundheitssystem wäre gut beraten, nach der Krise nicht in den üblichen Modus Operandi zu verfallen, sondern kritisch zu hinterfragen, warum etwa die Digitalisierung nicht stärker Einzug gehalten hat und warum organisatorische Innovationen nicht oder nur im geringen Maße intrinsisch erfolgten. Darüber hinaus würde eine regelmäßige Evaluierung zu einem lernenden System beitragen, wie wir das aus anderen regulierten Sektoren kennen. So könnte der Sachverständigenrat zur Begutachtung der Entwicklung im Gesundheitswesen etwa alle zwei Jahre auch den Stand und die Entwicklung des Wettbewerbs im Gesundheitswesen begutachten. Der Risikostrukturausgleich könnte regelmäßig, etwa alle drei bis fünf Jahre, unabhängig, also außerhalb des Bundesamts für Soziale Sicherung, evaluiert werden.

Ein wesentlicher Aspekt ist dabei die Systematik des RSA, der zu einem „nachhaltigen" RSA weiterentwickelt werden sollte, der auch die langfristigen Auswirkungen von Entscheidungen der Krankenkassen berücksichtigt. Die Monopolkommission hat dazu vorgeschlagen, die Häufigkeit von Erkrankungen – die Inzidenzrate – im RSA mit zu berücksichtigen (Monopolkommission, 2017). So würde eine Krankenkasse, die ihren Versichertenstamm insgesamt besser versorgt bzw. behandelt, mehr Mittel aus dem RSA bekommen. Denkbar wäre auch, den Zeithorizont des RSA zu erweitern, so dass eine Krankenkasse zwar weiterhin jährlich die erwarteten Ausgaben erhält, diese aber zu einem Stichtag für mehrere Jahre, beispielsweise zehn Jahre, errechnet und festgelegt werden. Eine Behandlung einer versicherten Person, welche die Kosten für die nächsten zehn Jahre senkt, würde sich für die Krankenkasse dann auch finanziell lohnen.

Prof. Achim Wambach, Ph. D., ist Präsident des ZEW — Leibniz-Zentrums für Europäische Wirtschaftsforschung in Mannheim.


Literatur

Athey, S., M. Kremer, C. Snyder und A. Tabarrok (2020), In the Race for a Coronavirus Vaccine, We Must Go Big. Really, Really Big, The New York Times, 4. Mai.
Bertschek, I., M. Polder und P. Schulte (2017), ICT and Resilience in Times of Crisis: Evidence from Cross-Country Micro Moments Data, ZEW Discussion Paper, 17–030, https://ftp.zew.de/pub/zew-docs/dp/dp17030.pdf (27. Juli 2021).
Castillo J C Ahuja A Athey S Baker A Budish E Chipty T Glennerster R Kominers S D Kremer M Larson G Lee J Prendergast C Snyder C M Tabarrok A Tan B J Więcek W Market Design to Accelerate COVID-19 Vaccine Supply Science 2021 371 6534 1107 1109 10.1126/science.abg0889 33632897
Cramton P Stoft S A Capacity Market that Makes Sense The Electricity Journal 2005 18 7 43 54 10.1016/j.tej.2005.07.003
Delacrétaz, D., S. D. Kominers und A. Teytelboym (2020), Matching Mechanisms for Refugee Resettlement, Working Paper.
Drösler, S., E. Garbe, J. Hasford, I. Schubert, V. Ulrich, W. van de Ven, A. Wambach, J. Wasem und E. Wille (2017), Sondergutachten zu den Wirkungen des morbiditätsorientierten Risikostrukturausgleichs.
Drösler, S., E. Garbe, J. Hasford, I. Schubert, V. Ulrich, W. van de Ven, A. Wambach, J. Wasem und E. Wille (2018), Gutachten zu den regionalen Verteilungswirkungen des morbiditätsorientierten Risikostrukturausgleichs.
Fabra, N., M. Motta und M. Peitz (2020), Preparing for the Next Crisis: How to Secure the Supply of Essential Goods and Services, Centre for Economic Policy Research.
Graumann, S. und I. Bertschek (2017), Monitoring-Report Wirtschaft DIGITAL 2017-Kompakt, ZEW-Gutachten und Forschungsberichte.
Gretschko, V. und M. Ott (2021), Ein flexibles Vergütungskonzept für Mediziner hilft, Herdenimmunität gegen COVID-19 zu erreichen, ZEW policy brief, 3.
Gretschko V A Procurement Mechanism to Assign Refugee Quotas Journal of Institutional and Theoretical Economics 2019 175 1 53 57 10.1628/jite-2019-0008
Gretschko, V. und A. Wambach (2021), Wie Prämien die Corona-Impfungen deutlich beschleunigen könnten, Handelsblatt, 31. Januar.
Kremer, M., J. Levin und C. M. Snyder (2020), Advance Market Commitments: Insights from Theory and Experience, AEA Papers and Proceedings, 110.
Kübler, D. (2021), Die Vergabe von Impfterminen nach dem Windhundprinzip ist verschwenderisch und unfair, Handelsblatt, 28. Januar.
Kübler D Ockenfels A Überkreuznierenspenden in Deutschland? Medizinrecht 2020 38 2 89 94 10.1007/s00350-020-5455-9
Monopolkommission (2017), 75. Sondergutachten: Stand und Perspektiven des Wettbewerbs im deutschen Krankenversicherungssystem, Nomos Verlag.
Monopolkommission (2018), 1. Policy Brief: Vergütung für Apotheken jetzt reformieren, Nomos Verlag.
Roth A E The Evolution of the Labor Market for Medical Interns and Residents: A Case Study in Game Theory Journal of Political Economy 1984 92 6 991 1016 10.1086/261272
Roth A E Sönmez T Utku Ünver M Kidney exchange The Quarterly Journal of Economics 2004 119 2 457 488 10.1162/0033553041382157
Wissenschaftlicher Beirat beim Bundesministerium für Wirtschaft und Energie (2021), Digitalisierung in Deutschland — Lehren aus der Corona-Krise, Gutachten des Wissenschaftlichen Beirats beim Bundesministerium für Wirtschaft und Energie.
